# Firearm Violence Exposure and Functional Disability among Black Men and Women in the United States

**DOI:** 10.1007/s11524-024-00866-8

**Published:** 2024-05-16

**Authors:** Daniel C. Semenza, Nazsa S. Baker, Christopher St. Vil

**Affiliations:** 1 Department of Sociology, Anthropology, and Criminal Justice, Rutgers University – Camden, 405-7 Cooper Street, Camden, NJ 08102 USA; 2 Department of Urban-Global Public Health, Rutgers University – New Brunswick, New Brunswick, NJ USA; 3 New Jersey Gun Violence Research Center, Rutgers University, New Brunswick, NJ USA; 4 School of Social Work, University at Buffalo, Buffalo, NY USA

**Keywords:** Firearm violence, Functional health, Disability, Sex differences, Black Americans

## Abstract

This study investigates the relationship between firearm violence exposure and functional health among Black adults in the United States (US). We examined associations between different forms of firearm violence exposure (direct, indirect, and community) and functional health with particular attention to differences across sex groups. We used survey data from a nationally representative sample of 3015 Black adult Americans to analyze associations between types of firearm violence exposure and four aspects of functional disability including: the ability to concentrate, walk/use stairs, dress/bathe, and run errands among males and females. The findings indicate notable disparities in exposure and health outcomes based on the exposure type and cumulative exposure to violence. Among males, functional disability was associated most closely with community violence exposure, while direct threats of firearm violence were most consequential for functional health among females. High cumulative exposure to firearm violence was linked to significant risks to functional health, particularly among females. The results shed light on sex differences in the repercussions of firearm violence exposure and emphasize its implications for daily functioning and health. This study contributes to the understanding of the multifaceted impacts of firearm violence on functional well-being and highlights the need for inclusive and culturally sensitive healing approaches based in community settings. There is a critical need for heightened awareness and strategies to enhance the well-being of those disproportionately affected by firearm violence in the US.

Firearm violence is an enduring public health crisis in the United States (US), leading to significant physical injury, psychological harm, and premature death. Approximately 85,000 people suffer from firearm injuries each year and firearm injuries are the leading cause of death among children and adolescents ages 1 to 19 years old [1–3]. Those who survive firearm injuries are almost twice as likely to suffer from long-term disability [4], costing Americans $48 to $175 billion annually [5]. Non-fatal firearm injury is a chronic, recurrent problem and occurs up to four times as frequently as firearm homicide [1, 6].

Black Americans experience a disproportionate burden of violent injury and exposure to firearm violence in the US [7–9]. Firearm-related violence is the leading cause of death and disability among Black boys and men ages 15–34 and the second leading cause of death for Black girls and women ages 15–24 [7]. The majority of Black men and women exposed to firearm violence reside in low-income urban communities, placing them at high risk for repeated exposure [10] and resultant ongoing physical and psychological health challenges [11]. Black men disproportionately experience direct firearm violence victimization (being shot or personally threatened with a firearm) while Black women often experience indirect exposure as co-survivors via the shooting or killing of a friend or loved one [12].

Black Americans are far more likely than those in other racial groups to experience community firearm violence exposure by residing in neighborhoods where shootings regularly occur [13, 14]. Chronic community violence exposure is associated with a host of health problems including depression, anxiety, and suicidal ideation [13, 15], poorer physical health [16–19], desensitization [11, 20], and chronic illness [21, 22]. Communities with high rates of shootings also experience poorer health behaviors related to obesity, smoking, physical inactivity, and sleep [16, 23, 24]. Taken together, Black Americans disproportionately experience numerous types of firearm violence exposure detrimental to mental, physical, and behavioral well-being.

## Firearm Violence Exposure and Functional Health

Both direct and indirect firearm violence exposure shape how people function on a day-to-day basis and carry out activities of daily living [17, 25]. Survivors with firearm-related injuries often contend with physical complications that make daily living far more difficult [26, 27]. Some firearm violence survivors become paralyzed and confined to a wheelchair while others temporarily need assistive technology for mobility such as crutches, canes, or walkers [28]. Physical injuries from a firearm injury often lead to long-term chronic pain [29], which impacts daily activities like using the stairs, running errands, or cooking a meal. Beyond physical complications, individuals who have been shot or threatened with a firearm often cope with psychological consequences including post-traumatic stress disorder (PTSD), depression, and substance abuse [30–32]. The many health complications that stem from firearm violence exposure are likely to create additional functional limitations for those who survive.

Exposure to firearm violence also impacts the everyday functioning of co-survivors and those living in high-violence communities. Family and friends of those who are shot and/or killed experience greater mental health needs after a shooting [33–35], including PTSD and other symptoms of trauma [36]. Caregivers and family members are often terrified of being victimized themselves, preventing them from going outside and spending time in their communities [37]. Fear and hypervigilance in the wake of a loved one’s attack is also likely to shape how people conduct daily activities like running errands, concentrating on everyday tasks, or traveling to the doctor’s office [38]. Similarly, many community violence interventionists experience significant secondary trauma that can greatly hamper their ability to conduct their work and function on a daily basis [39].

Despite a growing body of literature on the health implications of firearm violence exposure, there remain few studies about how specific types of firearm violence exposure (e.g., direct, secondary, community) correspond to various aspects of functional health. Further, there has been no research to our knowledge that assesses how *cumulative* firearm violence exposure is associated with functional well-being. Finally, research remains limited on differences in firearm violence exposure between men and women and the implications for functional health, despite prior work indicating that divergent forms of direct and indirect violence are particularly salient for the well-being of men and women [12, 40, 41]. To address these limitations, we analyzed the association between firearm violence exposures and functional health in a nationally representative sample of Black men and women in the US.

## Methods

### Sample

This study uses survey data from 3015 Black adults (18 +) living in the US, collected in April and May 2023. The authors designed the survey and it was disseminated by Ipsos Public Affairs, an international market research and data collection firm. The study sample was drawn from Ipsos’ KnowledgePanel (KP), a nationally representative online panel designed to represent the full population of the US. KP panel members are randomly recruited through a probability-based approach that uses address-based sampling (ABS) methods. KP panel members filled out an initial screener survey to determine racial identity and were invited to complete the present survey if they identified as Black or African American.

We specifically focused on Black Americans in this survey for two reasons. First, as noted above, Black Americans are much more likely than any other racial group in the US to be exposed to high levels of multiple forms of firearm violence [8, 9, 13, 14]. Second, historical and structural racism disproportionately exposes Black Americans to numerous intersecting harms related to deprivation, discrimination, and health inequality that shape access to care and functional health [42]. We thus chose to be intentional in our focus on Black Americans here given the unique context of everyday life likely to influence significant inequities in both our exposures and outcomes of interest.

Using geodemographic benchmarks from the American Community Survey (CS) and the Census Burau’s Current Population Survey (CPS) on the full KP sample, the study’s specific sample was selected using a probability-proportional-to-size (PPS) procedure to ensure the sample is demographically balanced and representative of Black adults in the US. An iterative proportional fitting (raking) procedure was used to produce the final design weights and ensure proper distribution for the 18 + population across sex, census region, metropolitan status, education, and household income benchmarks. The weights were trimmed and scaled to add up to the total number of qualified Black respondents. Please see Appendix Table 4 for full information on the demographic distributions of the benchmarks for our sample. The study was fully approved by the International Review Board at Rutgers University.

### Measures

#### Functional Health

The main outcome variable measures four aspects of functional disability derived from the American Community Survey [43, 44]. We measured *concentration* with the question, “Because of a physical, mental, or emotional condition, do you have serious difficulty concentrating, remembering, or making decisions?” Difficulty with *walking/stairs* was measured by asking, “Do you have serious difficulty in walking or climbing stairs?” We measured difficulty *dressing/bathing* with the question, “Do you have difficulty dressing or bathing?” and difficulty doing everyday tasks like *running errands* by asking, “Because of a physical, mental, or emotional condition, do you have difficulty doing errands alone such as visiting a doctor’s office or shopping?” All responses were binary (0 = no; 1 = yes). These four items were treated as discrete outcome measures and also added up to create a variety scale of *total disability* (range 0–4).

#### Firearm Violence Exposure

The main exposures included four items related to firearm violence exposure [45]. We asked respondents if they had (1) ever been threatened with a firearm by another person or (2) been shot on purpose by another person with a firearm. We also asked, (3) “Do you personally know someone, such as a friend or family member, who has been shot on purpose by another person with a firearm? Finally, we asked, (4) “Have you ever witnessed or heard about someone being shot intentionally by another persona with a firearm in your neighborhood?” All responses were coded as binary answers (0 = no; 1 = yes). We added these four items to create a measure of *cumulative firearm violence exposure*. Due to a very small number of respondents who experienced all four types of exposure, we combined the final two responses into a single category as “three or more types” of exposure.

#### Covariates

All models account for pertinent covariates including self-rated health (poor, fair, good, very good, excellent), age category (18–29, 30–44, 45–59, 60 +), education level (no high school, high school degree, some college, Bachelors or more), household income category (< $24,999, $25 K to $74,999, $75 K to $149,999, $150 K +), marital status (married, widowed, divorced, separated, never married), employment status (working full time, working part time, not working), health insurance (yes, no), metro area residence (yes, no), and region of residence (Northeast, Midwest, South, West).

### Analytic Strategy

 We used a four-part analytic strategy. We first generated weighted descriptive statistics for the full sample. We then assessed bivariate differences between males and females for individual and cumulative firearm violence exposure and functional disability. Third, we ran a series of multivariate regressions on male and female subsamples to assess the influence of individual firearm violence exposures on total and individual types of functional disability. Finally, we ran a series of regression models on the two subsamples to assess the association between cumulative firearm violence exposure and all functional disability outcomes. We used negative binomial regression for the total disability outcome given the over-dispersed distribution of the count measure and logistic regression for each individual disability type given their binary distributions. We used listwise deletion to account for a small number of cases in the full sample with missing variables (*N* = 88; 2.9%). We conducted all analyses in Stata 17.

## Results

Table 1 depicts the weighted summary statistics for the full sample. The most common type of firearm violence exposure was knowing a family member or friend who had been shot (41%) followed by witnessing or hearing about a shooting in one’s community (38%). The majority of respondents were exposed to at least one type of firearm violence (59%) and 12% of the sample had been exposed to three or more types of firearm violence. The most common functional disability was problems concentrating (16%) followed by issues walking or taking the stairs (12%). On average, respondents reported 0.42 functional disabilities on a scale of 0–4.

**Table 1:** Weighted descriptive statistics for full sample (*N* = 3015)

	*N*	%		*N*	%
Firearm violence exposure types			Education		
Threatened w/ firearm	649	22	No HS	177	6
Shot w/ firearm	80	3	HS degree	1128	37
Family/friend shot	1237	41	Some college	928	31
Witnessed/heard about shooting	1138	38	Bachelors or more	782	26
Cumulative firearm violence exposure			Household income		
None	1230	41	< $24,999	621	21
One	789	27	$25,000 to $74,999	1163	39
Two	604	20	$75,000 to $149,999	831	28
Three or more	300	12	$150,000 +	399	13
Functional disabilities			Marital status		
Concentration	474	16	Married	1079	36
Walking/stairs	368	12	Widowed	149	5
Dressing/bathing	136	5	Divorced	364	12
Errands	280	9	Separated	66	2
Female	1646	55	Never married	1357	45
Self-rated health			Employment status		
Poor	75	2	Working full time	1596	53
Fair	607	21	Working part time	340	11
Good	1262	42	Not working	1079	36
Very good	789	27	Health insurance	2697	90
Excellent	264	9	Metro area residence	2756	91
Age			Region of residence		
18–29	562	19	Northeast	513	17
30–44	972	32	Midwest	484	16
45–59	728	24	South	1700	57
60 +	754	25	West	317	10
				M	SD
			Total functional disabilities (0–4)	0.42	0.83
			# of children living at home	0.64	1.07

**Fig. 1:**
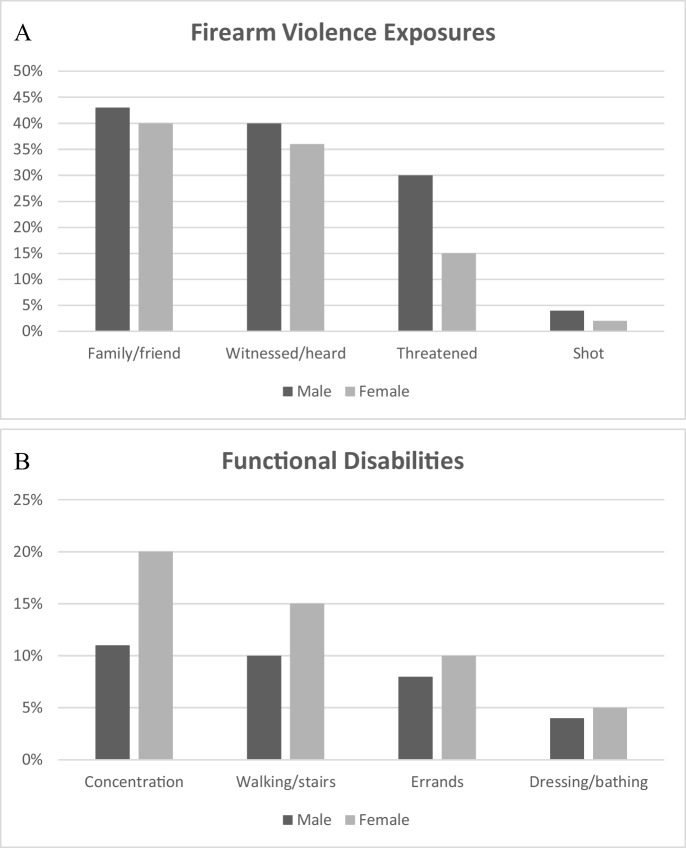
Individual firearm violence exposure types and functional disabilities by sex group Panel **A** and Panel **B**

Figure 1 illustrates bivariate sex differences in individual firearm violence exposures and functional disabilities. In general, men indicated slightly higher levels of knowing a family or friend that has been shot (43% vs 40%) and witnessing/hearing about a shooting (40% vs 36%). Men were two times as likely to report being threatened with a firearm (30% vs 15%) and being shot with a firearm (4% vs 2%). Women experienced higher levels of functional disability for problems concentrating (20% vs 11%) and walking/stairs (15% vs 10%). Women also reported slightly higher levels of functional disability related to running errands (10% vs 8%) and self-care (dressing/bathing; 5% vs 4%).

**Fig. 2:**
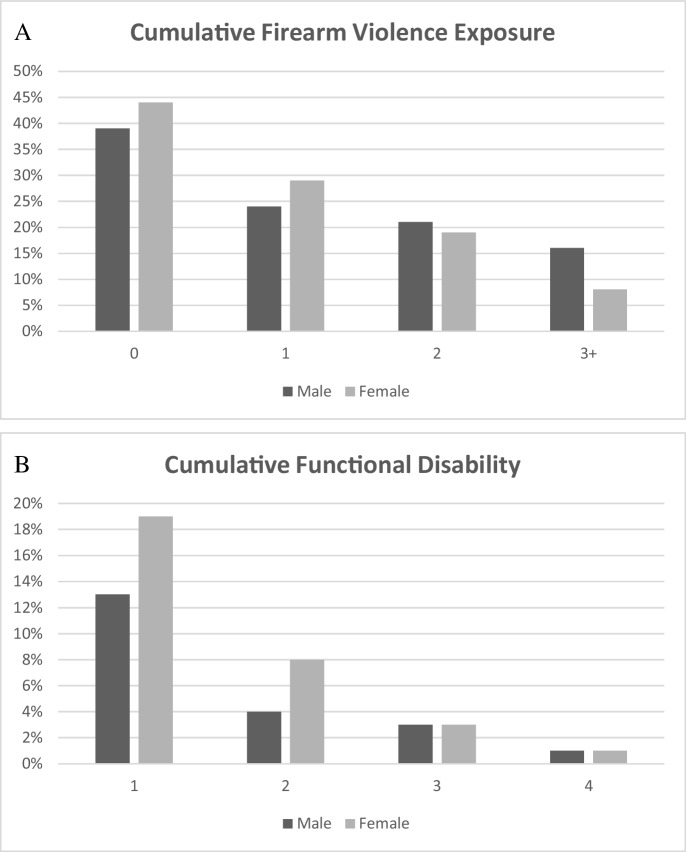
Cumulative firearm violence exposure and functional disability by sex group Panel **A** and Panel **B**

Figure 2 depicts bivariate sex differences for cumulative firearm violence exposure and functional disability. In general, women reported higher levels of exposure to zero and one type of firearm violence. Men reported slightly higher cumulative exposure of two types (21% vs 19%) and double the rate of exposure for women to three or more types of firearm violence (16% vs 8%). For cumulative functional disability, women reported higher rates of one (19% vs 13%) and two (8% vs 4%) impairments than men. However, rates of experiencing three (3%) and all four (1%) impairments were equal among sex groups.

Table 2 provides the results for the association between individual firearm violence exposures and functional disability by sex group. For men, witnessing or hearing about a shooting was associated with greater risk for total disability (IRR = 1.53; *p* < 0.016), problems concentrating (OR = 2.12, *p* < 0.037), walking and taking the stairs (OR = 2.39, *p* < 0.005), and dressing/bathing (OR = 2.66, *p* < 0.008). No other type of firearm violence exposure was related to any disability outcome among men. For women, being threatened with a firearm was associated with total disability (IRR = 1.48, *p* < 0.000) as well as problems with concentration (OR = 1.75, *p* < 0.020), walking/stairs (OR = 2.00, *p* < 0.002), and running errands (OR = 2.29, *p* < 0.002). Additionally, knowing a family member or friend who had been shot was associated with significantly greater odds of difficulty running errands for women only (OR = 1.81, *p* < 0.025).

**Table 2:** Individual firearm violence exposures and functional disability by sex group

	Total disability					Concentration					Walking/stairs				
	IRR	SE	*p*	CI		OR	SE	*p*	CI		OR	SE	*p*	CI	
*Men (N* = *932)*															
Threatened	1.08	0.22	0.705	0.73	1.61	1.02	0.37	0.953	0.51	2.06	0.95	0.32	0.888	0.50	1.82
Shot	0.90	0.29	0.742	0.48	1.68	0.98	0.61	0.969	0.29	3.33	1.26	0.62	0.639	0.48	3.29
Family/friend shot	1.10	0.20	0.596	0.77	1.59	1.65	0.60	0.162	0.82	3.35	0.87	0.29	0.682	0.46	1.66
Witnessed/heard	1.53*	0.27	0.016	1.08	2.16	2.12*	0.76	0.037	1.05	4.30	2.39**	0.74	0.005	1.30	4.41
*Women (N* = *1995)*															
Threatened	1.48***	0.15	0.000	1.21	1.82	1.75*	0.42	0.020	1.09	2.80	2.00**	0.46	0.002	1.28	3.13
Shot	1.66	0.67	0.210	0.75	3.68	1.92	1.00	0.214	0.69	5.34	2.95	2.27	0.160	0.65	13.37
Family/friend shot	1.21	0.13	0.067	0.99	1.49	1.27	0.27	0.260	0.84	1.91	1.22	0.25	0.337	0.81	1.82
Witnessed/heard	1.14	0.11	0.208	0.93	1.38	1.19	0.24	0.384	0.80	1.78	1.25	0.23	0.229	0.87	1.81
	Dressing/bathing					Errands									
	OR	SE	*p*	CI		OR	SE	*p*	CI						
*Men (N* = *932)*															
Threatened	1.26	0.50	0.565	0.57	2.76	0.97	0.35	0.936	0.48	1.95					
Shot	1.65	1.06	0.436	0.47	5.81	0.63	0.44	0.505	0.16	2.47					
Family/friend shot	0.86	0.34	0.695	0.40	1.85	1.07	0.37	0.846	0.54	2.11					
Witnessed/heard	2.66**	0.98	0.008	1.29	5.50	1.51	0.54	0.245	0.75	3.04					
*Women (N* = *1995)*															
Threatened	1.74	0.55	0.080	0.94	3.22	2.29**	0.60	0.002	1.37	3.82					
Shot	5.16	4.51	0.061	0.93	28.64	2.04	1.66	0.379	0.42	10.04					
Family/friend shot	1.29	0.37	0.371	0.74	2.28	1.81*	0.48	0.025	1.08	3.05					
Witnessed/heard	1.12	0.35	0.717	0.61	2.06	1.13	0.27	0.603	0.71	1.82					

All models control for self-rated health, age, education, household income, marital status, employment status, number of children living in the home, insurance status, metro area residence, and US region *p* ≤ 0.001 *** *p* ≤ 0.01 ** *p* ≤ 0.05

Table 3 depicts the multivariate results for cumulative firearm exposure and all functional disability outcomes in both sex groups. For men, exposure to three or more types of firearm violence was associated with greater total disability (IRR = 1.83, *p* < 0.011). For individual disabilities, exposure to both one (OR = 2.74, *p* < 0.028) and three or more types (OR = 3.94; *p* < 0.002) was associated with a much greater risk of problems with concentration. Cumulative exposure was not associated with any other individual functional disability among men. For women, exposure to three or more types of firearm violence was associated with substantially higher risk for total disability (IRR = 2.37, *p* < 0.000) and all types of individual disability. Odds ratios ranged from 3.33 for problems dressing/bathing (*p* < 0.010) to 5.83 for problems running errands (*p* < 0.000).

**Table 3:** Cumulative firearm violence exposure and functional disability by sex group

	Total disability					Concentration					Walking/stairs				
	IRR	SE	*p*	CI		OR	SE	*p*	CI		OR	SE	*p*	CI	
*Men (N* = *932)*															
One type	1.32	0.29	0.210	0.86	2.03	2.74*	1.25	0.028	1.11	6.73	1.25	0.49	0.572	0.58	2.72
Two types	1.36	0.32	0.194	0.85	2.17	2.16	0.95	0.081	0.91	5.14	2.03	0.84	0.087	0.90	4.58
Three or more types	1.83**	0.43	0.011	1.15	2.91	3.94***	1.70	0.002	1.69	9.20	1.83	0.71	0.122	0.85	3.93
*Women (N* = *1995)*															
One type	1.11	0.14	0.421	0.86	1.43	1.41	0.33	0.141	0.89	2.21	0.99	0.25	0.963	0.60	1.64
Two types	1.30	0.16	0.036	1.02	1.66	1.25	0.32	0.381	0.76	2.06	1.57*	0.36	0.050	1.00	2.46
Three or more types	2.37***	0.38	0.000	1.73	3.26	3.72***	1.05	0.000	2.13	6.48	3.71***	1.29	0.000	1.87	7.33
	Dressing/bathing					Errands									
	OR	SE	*p*	CI		OR	SE	*p*	CI						
*Men (N* = *932)*															
One type	0.73	0.36	0.523	0.27	1.94	0.91	0.36	0.810	0.41	1.99					
Two types	1.39	0.71	0.523	0.51	3.80	0.96	0.44	0.924	0.38	2.38					
Three or more types	2.58	1.29	0.058	0.97	6.86	1.57	0.71	0.315	0.65	3.80					
*Women (N* = *1995)*															
One type	0.81	0.31	0.591	0.38	1.74	1.18	0.43	0.639	0.59	2.39					
Two types	1.33	0.59	0.516	0.56	3.19	1.83	0.58	0.054	0.99	3.40					
Three or more types	3.33*	1.54	0.010	1.34	8.27	5.83***	2.01	0.000	2.97	11.44					

All models control for self-rated health, age, education, household income, marital status, employment status, number of children living in the home, insurance status, metro area residence, and US region *p* ≤ 0.001 *** *p* ≤ 0.01 ** *p* ≤ 0.05

## Discussion

In this study, we examined associations between specific types of firearm violence exposure and various aspects of functional health among a sample of Black adults in the US. We also explored differences in these relationships across sex groups. The findings illustrate deleterious, varied impacts of firearm violence exposure on functional well-being, with particular harms related to cumulative exposure. We found that firearm violence exposures influence functional health differently among men and women. Specifically, witnessing or hearing about someone getting shot (i.e., community violence) was associated with poorer functional health among men while being directly threatened with a firearm was most consequential for daily functional activities among women. Experiencing three or more cumulative forms of firearm violence exposure was especially linked to poorer total functional disability and issues with concentration among men, while the same high level of exposure among women was related to substantially higher risk for all types of functional disability.

The distinction in the findings between men and women is notable, though we are cautious not to interpret differences in individual incidence and odds ratios across subsamples. Although men and women experienced relatively similar rates of witnessing or hearing about a shooting, this type of community exposure was only associated with functional health among men. Conversely, men are much more likely to report being threatened by a firearm yet this exposure was only significantly associated with functional health among women. These findings cohere with prior literature suggesting indirect forms of firearm violence exposure may be especially salient for well-being among men while direct violence is more affecting for women [12, 40]. Research suggests that although men and boys are more likely to report potentially traumatic events such as being threatened with a firearm, women and girls show an elevated risk of developing symptomology of post-traumatic stress disorder (PTSD) as a result of such events [46]. This gendered dynamic may operate similarly for aspects of functional well-being.

Men may hear about or witness someone being shot more often due to greater participation within social networks of other men who engage in firearm violence [47, 48]. The density of these networks may lead to disproportionate community firearm violence exposure among men [49–51]. Homicide rates in the US are highest among non-Hispanic Black males aged 15–44 and shootings most commonly occur between acquaintances where victims know the perpetrator [52]. Although our bivariate results suggest that men only experience community violence exposure slightly more than women, the exposure may be much more pervasive among men in concentrated social networks with greater implications for functional health than for women. We were unable to measure the frequency and recency of exposure for different types of firearm violence, so clarifying these aspects should be a priority for future researchers.

For women, the finding that being threatened with a firearm is linked to much poorer functional health may relate to intimate partner violence victimization [53, 54]. Prior research suggests that men are more likely to experience violent victimization by a friend or known associate while women more often experience such violence at the hands of a current or former partner [52]. The significant relationship between knowing a family member or friend who has been shot with greater difficulty running errands in our findings suggests that vicarious trauma may be particularly damaging to women’s sense of safety and security, reducing the capacity to conduct daily activities outside of the home [55]. This dynamic may be especially acute in communities where public spaces are dominated by men who sexualize interactions with women and act as potential threats [56]. Choosing not to run errands or go outside of the home may reflect a strategy of withdrawing from public life, particularly in the wake of losing a loved one in a violent attack [57].

The enhanced influence of cumulative violence among women across all types of functional health exhibited in our findings is consistent with research that operationalizes such experiences as “cumulative co-victimization” [41]. From this perspective, cumulative vicarious trauma “disrupts Black women’s sensibilities about the social environment, leaving many of them cynical and distrustful of their neighborhoods and its residents” [41, p.8]. Black women who experience multiple forms of firearm violence, directly and indirectly, may be unable to engage in many of the activities that comprise daily life. For instance, it may be much harder to concentrate on basic tasks if a person is contending with symptoms of anxiety or depression stemming from the threat of pervasive violence [58]. On the other hand, it is likely much more difficult to take the stairs or dress/bathe one’s self when dealing with a physical limitation brought about by high levels of violence exposure [18]. Our results show the largest effects for running errands, suggesting that women may be unable or perhaps unwilling to leave the home in the face of significant danger.

These findings have implications for improving service provision and support for those exposed to firearm violence to address functional health, particularly among those who experience cumulative exposure. For instance, men living in communities where witnessing or hearing about firearm violence is a regular occurrence may not discuss these experiences with healthcare professionals or close loved ones, especially if these instances are normalized in the local community. Men may also not feel comfortable speaking with a therapist or family member if they have been vicariously exposed to firearm violence, seeing an effort to cope as a sign of weakness or threat to their own self-conceptualization of masculinity [59]. On the other hand, women who are directly exposed to instances of intimate partner violence may be unable to turn to formal or informal support networks out of shame or fear of retaliation [60, 61]. It is thus crucial that culturally congruent opportunities for coping and healing are made readily available to those exposed to firearm violence to avoid broader harms to everyday health. For instance, numerous community violence intervention programs, such as Roca, integrate trauma-informed cognitive behavioral therapy (CBT) into their curriculum, which has been shown to improve emotional regulation, enhance interpersonal relationships, and increase employment opportunities [62–64]. Deployment of informal CBT in a community-based setting can potentially provide the additional benefit of buffering against functional disabilities among those who experience gun violence exposure, particularly disabilities related to concentration shown here to be meaningfully associated with gun violence exposure for both men and women.

Research shows that Black Americans exposed to firearm violence employ spiritual coping and meaning-making as strategies to process pain and reduce fear [12, 65, 66]. Black Americans are more likely to turn to communal and collectivistic approaches to coping with stress and loss rather than seek support from a formal support network or healthcare resource (e.g., counseling with a therapist). A sociocultural responsive model of coping for African Americans exposed to firearm violence therefore entails opportunities for coping and healing that account for broader cultural traumas, stigmatization, and historical harms from law enforcement and traditional healthcare systems [67]. Provision of victim services in both therapeutic and community-based settings for Black Americans affected by firearm violence should aim to integrate culturally tailored interventions, potentially through collaborations with pastoral counseling centers, clergy, and spiritual leaders, to provide services that offer the greatest benefit and opportunity for protection against further health harms [68].

Additionally, hospital-based violence intervention programs (HVIPs) offer services to aid in long-term well-being among direct gun violence victims while ensuring that survivors do not engage in retaliatory actions once they leave the hospital [69]. However, HVIPs are frequently designed to provide the most robust services for young men given their disproportionate involvement in gun violence with far less attention to women who are victims of gun violence [70, 71]. Further, HVIPs are largely geared towards direct victims of violence (i.e., those who have been shot). Our results suggest that HVIPs should be equipped to provide wraparound services to both men and women, while also offering service opportunities for secondary survivors such as friends and family members of direct victims. A holistic approach that provides direct services for all of those touched by a given shooting may aid in reducing the risk of functional disabilities that develop in the wake of diverse forms of gun violence exposure.

There are certain limitations to this study that provide opportunities for future research. First, there are limitations to our exposure measures. For instance, our measure of community violence exposure combined witnessing and hearing about a shooting, which should be disaggregated in future studies [72, 73]. We were also only able to measure lifetime experiences of firearm violence exposure. Researchers should consider asking about the frequency of different types of firearm violence exposure while using multiple time anchors (e.g., exposure in the past year, 6 months, 2 weeks, etc.) to discern how proximity to exposure and its frequency shape health outcomes.

Second, the data are cross-sectional and our analyses preclude any causal claims. Longitudinal research is needed to properly understand how individual firearm exposures and cumulative experiences shape health and functional well-being over time. Finally, despite our intentional focus on Black Americans for this study, our findings are only generalizable to those adults who identified as Black or African American in the US. It will be important to replicate the analyses here to understand how firearm violence exposure shapes functional health and daily activities among other racial groups as well as children across these groups.

Despite these limitations, this is the first study to our knowledge that examines the relationship between firearm violence exposure and functional well-being alongside related sex disparities. We analyzed these dynamics specifically among Black adults, a group of Americans disproportionately exposed to firearm violence and its many collateral consequences. Efforts to reduce firearm violence are necessary to address health disparities throughout the country and our findings suggest the benefits might extend to functional well-being. Ultimately, preventing firearm violence exposure is critical to ensure that all people have the same opportunity to live meaningful, healthy lives.

## Data Availability

Data and related syntax are available upon request from the authors.

## Appendix 1

Table 4

**Table 4:** 18 + African American distribution benchmarks for design weights

Gender	Frequency	Percent
Male	15,975,616	45.97
Female	18,776,282	54.03
Age	Frequency	Percent
18–44	18,171,478	52.29
45–59	8,165,840	23.5
60 +	8,414,579	24.21
Sex/age	Frequency	Percent
18–44 male	8,626,014	24.82
18–44 female	9,545,464	27.47
45–59 male	3,747,000	10.78
45–59 female	4,418,840	12.72
60 + male	3,602,602	10.37
60 + female	4,811,977	13.85
Race	Frequency	Percent
African American only	34,358,555	98.87
Both	393,342.6	1.13
Region	Frequency	Percent
Northeast	5,988,446	17.23
Midwest	5,617,959	16.17
South	19,544,336	56.24
West	3,601,157	10.36
MSA status	Frequency	Percent
Non-Metro	2,901,957	8.35
Metro	31,849,941	91.65
Education	Frequency	Percent
LHS/HS	15,664,113	45.07
Some college	10,359,643	29.81
Bachelor or higher	8,728,141	25.12
Income	Frequency	Percent
Under $25,000	6,930,017	19.94
$25,000–$49,999	7,219,110	20.77
$50,000–$74,999	6,005,875	17.28
$75,000–$99,999	4,270,889	12.29
$100,000–$149,999	5,268,908	15.16
$150,000 and over	5,057,099	14.55
